# Supplementary material for: Splice-Junction-Based Mapping of Alternative Isoforms in the Human Proteome
Source: Cell Rep. Author manuscript; Available in PMC 2020 Jan 15. (PMC6961840; doi:10.1016/j.celrep.2019.11.026)
Supplement: 3 [file NIHMS1546469-supplement-3.zip › DF2/PXD0010154/Lung-240-P12111-TLSGTPEESKR.pdf]

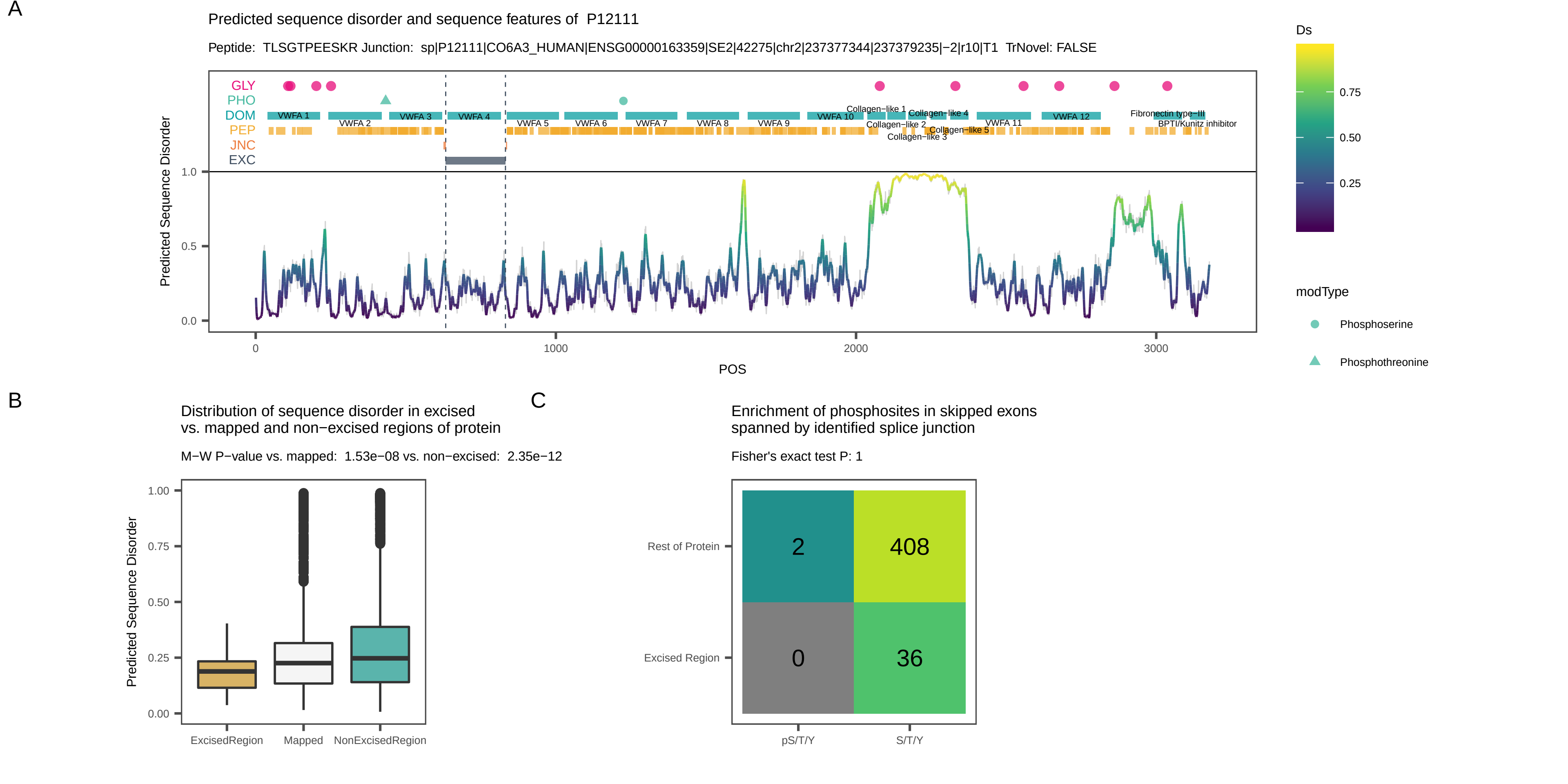
A
Predicted sequence disorder and sequence features of P12111
Peptide: TLSGTPEESKR Junction: sp|P12111|CO6A3_HUMAN|ENSG00000163359|SE2|42275|chr2|237377344|237379235|−2|r10|T1 TrNovel: FALSE
GLY
PHO
DOM
PEP
JNC
EXC
VWFA 1
VWFA 2
VWFA 3
VWFA 4
VWFA 5
VWFA 6
VWFA 7
VWFA 8
VWFA 9
VWFA 10
VWFA 11
VWFA 12
Collagen−like 1
Collagen−like 2
Collagen−like 3
Collagen−like 4
Collagen−like 5
Fibronectin type−III
BPTI/Kunitz inhibitor
Predicted Sequence Disorder
1.0
0.5
0.0
0
1000
2000
3000
POS
Ds
0.75
0.50
0.25
modType
Phosphoserine
Phosphothreonine
B
Distribution of sequence disorder in excised
vs. mapped and non−excised regions of protein
M−W P−value vs. mapped: 1.53e−08 vs. non−excised: 2.35e−12
Predicted Sequence Disorder
1.00
0.75
0.50
0.25
0.00
ExcisedRegion
Mapped
NonExcisedRegion
C
Enrichment of phosphosites in skipped exons
spanned by identified splice junction
Fisher's exact test P: 1
Rest of Protein
Excised Region
2
408
0
36
pS/T/Y
S/T/Y
